# Development of a multicomponent intervention to increase parental vaccine confidence and young people’s access to the universal HPV vaccination programme in England: protocol for a co-design study

**DOI:** 10.1136/bmjopen-2022-062050

**Published:** 2022-04-06

**Authors:** Harriet Fisher, Tracey Chantler, Sarah Denford, Adam Finn, Matthew Hickman, Sandra Mounier-Jack, Marion Roderick, Leanne Tucker, Julie Yates, Suzanne Audrey

**Affiliations:** 1 National Institute for Health Research Health Protection Research Unit (NIHR HPRU) in Behavioural Science and Evaluation (BSE), University of Bristol, Bristol, Bristol, UK; 2 National Institute for Health Research Health Protection Research Unit (NIHR HPRU) in Vaccinations and Immunisation, London School of Hygiene and Tropical Medicine, London, UK; 3 School of Cellular and Molecular Medicine, University of Bristol, Bristol, UK; 4 Department of Paediatric Immunology and Infectious Diseases, University Hospitals of Bristol and Weston NHS Foundation Trust, Bristol, UK; 5 Bristol Parent Carers, Bristol, UK; 6 Screening and Immunisation, South West, NHS England South, Taunton, UK

**Keywords:** EDUCATION & TRAINING (see Medical Education & Training), Protocols & guidelines, IMMUNOLOGY, QUALITATIVE RESEARCH, PUBLIC HEALTH

## Abstract

**Introduction:**

Persistent infection with HPV can result in cancers affecting men and, especially, women. Lower uptake exists by area and different population groups. Increasing parental confidence about, and adolescent access to, the universal HPV vaccination programme may help reduce inequalities in uptake. However, the evidence-base for interventions to address uptake for schools-based HPV vaccination programmes is currently lacking. This study protocol outlines how a multicomponent intervention to address this evidence gap will be codesigned with parents.

**Methods and analysis:**

The proposed research will be undertaken in localities covered by two immunisation teams in London and the south-west of England. The ‘person-based approach’ to intervention development will be followed. In the first phase, an exploratory qualitative study will be undertaken with key stakeholders (n=8) and parents (n=40) who did not provide consent for their adolescent child to be vaccinated. During the interviews, parents’ views on ways to improve parental confidence about, and adolescents’ access to, HPV vaccination will be sought. The findings will be used to inform the co-design of a preliminary plan for a targeted, multicomponent intervention. In the second phase, at least two parent working groups (n=8) will be convened and will work with creative designers to co-design communication materials aimed at increasing parents’ confidence in vaccination. At least two workshops with each parent group will be organised to obtain feedback on the intervention plan and communication materials to ensure they are fit for purpose. These findings will inform a protocol for a future study to test the effectiveness of the intervention at increasing HPV vaccination uptake.

**Ethics and dissemination:**

The National Health Services Research Ethics Service and London School of Hygiene & Tropical Medicine Observational / Interventions Research Ethics Committee provided approvals for the study (reference 22/SW/0003 & 26902, respectively). We will work with parent advisory groups to inform our dissemination strategy and co-present our findings (eg, at community events or through social media). We will disseminate our findings with academics and healthcare professionals through webinars and academic conferences, as well as peer-reviewed publications.

**Strengths and limitations of this study**

- Parents from a range of socioeconomic and ethnic backgrounds will be engaged to maximise the acceptability, feasibility and persuasiveness of the intervention.
- The recruitment strategy may miss key groups whose voices will be excluded.
- The communication materials developed may not address the information needs of all communities.

## Introduction

Human papillomavirus (HPV) is a common virus that can be transmitted sexually and cause a range of conditions affecting both women and men. These include precancerous lesions that may progress to cancers affecting the cervix, vulva, vagina, penis, anus and oral cavity. The three HPV vaccines—bivalent, quadrivalent and non-valent—all have proven safety profiles and are efficacious in producing strong immune responses when administered in early adolescence.^1 2^

In England, the universal HPV vaccination programme is offered to young people aged 12–13 years and is usually provided through a schools-based model of delivery. Although coverage of the programme is high, inequalities in uptake continue to persist with young people from minority ethnic groups and socioeconomically disadvantaged areas less likely to receive the HPV vaccine.^3 4^

There is increasing recognition that lower vaccine confidence (which is the trust in the effectiveness and safety of vaccines, and the healthcare system that delivers them) contributes to inequalities in uptake.^5^ Improving communication of evidence-based vaccine messages with families, and responding to misinformation circulating in social media and antivaccination activities, have been proposed as strategies to improve vaccine confidence.^6–8^ There is an increased urgency for action as reports show vaccination misinformation has proliferated during the COVID-19 pandemic.^9^

In schools-based vaccination programmes, information leaflets alongside forms requesting parental consent, are usually distributed by the school to parents or carers. This limits opportunities for immunisation teams to address lower vaccine confidence or to frame and target specific HPV vaccine messages to families with additional information needs.^10 11^ Communication materials that are tailored to address parents’ information needs are also required in order to effectively change how they think and feel about the HPV vaccine. Barriers to access, such as the availability of the HPV vaccine, may also need to be addressed.

We have co-produced with young people an educational package which is designed to be delivered in schools and answer young people’s questions about the HPV vaccine.^12^ We now plan to undertake complementary research by co-designing with parents who did not provide consent for their adolescent children a targeted, multicomponent intervention to increase parental vaccine confidence in, and adolescent access to, the HPV vaccine in England.

## Methods and analysis

This article outlines the protocol for a co-design study that is currently underway.

### Study setting

The study will take place in localities covered by two immunisation teams in London and the south-west of England.

### Study design

We are adopting a research approach which combines qualitative and co-design methods in order to investigate confidence and access issues and then develop an intervention. The 2021 guidance for complex intervention development and evaluation^13^ and the person-based approach to intervention planning and development^14^ inform the proposed research design. This will enable us to develop an appropriate theory-based, evidence-based and person-based framework to underpin the COMMUNICATE intervention.

Incorporating the views of the target users (parents who did not provide consent for their adolescent child to be vaccinated) throughout the development, design and testing processes increases the likelihood that the educational package will be acceptable, engaging, persuasive and easy to use. In turn, this is intended to promote engagement, implementation and ultimately, effectiveness.^15^

In line with the person-based approach,^14^ the intervention planning and development phase will involve the following interrelated stages: (i) collating and analysing evidence; (ii) developing guiding principles; (iii) undertaking a behavioural analysis; (iv) development of a preliminary logic model; (v) co-design of the intervention plan and communication materials and (vi) intervention refinement.

This will be informed by the findings from following planned research to be undertaken as part of this project: (i) a qualitative study with parents who did not provide consent for their adolescent children to receive the HPV vaccine and key stakeholders and (ii) co-design communication materials with parents and refiniement of the strategy for a targeted, multicomponent intervention to improve vaccine confidence and access.

### Patient and public involvement

The initial research idea builds on our previous qualitative research about the HPV vaccination programmes undertaken with families and immunisation nurses.^10 11^ We developed the study design further through patient and public involvement (PPI) in collaboration with two groups of parents of adolescent children at community groups serving disadvantaged populations in Bristol and London.

Discussions with parent advisory groups highlighted that parents valued clear information relating to side-effects, safety, effectiveness and contents of the vaccine. Two parents, who had refused vaccinations for their children, reported negative encounters with healthcare professionals and suggested a more empathetic approach or additional training of healthcare providers was needed to improve communication. Provision of additional information within the community setting (eg, community advocates, TV screens in General Practice surgeries, school websites) was also suggested to raise awareness. Both groups recognised barriers to access and suggested additional engagement of general practices and pharmacies to provide the HPV vaccine.

We also consulted young people from the Bristol Young Person’s Advisory Group (website: https://generationr.org.uk/bristol/). They recognised that parents could access unreliable vaccine information through the internet and social media outlets. Suggestions included modification of consent forms to link with websites providing parents with accurate, evidence-based information (eg, QR codes). These suggestions will be explored in greater depth during the interviews and workshops as part of the study.

PPI will be integrated at all stages of the research. We included a public co-applicant in the research team to ensure that both her views, and the perspectives of wider groups of parents, will be integrated from the early stages of the project, through to intervention development and dissemination of the study findings. We will also attempt to reach out to faith leaders or community advocates to be involved in the PPI group as part of the study.

The parents in the Bristol and London PPI advisory groups and the Bristol Young Person’s Advisory Group (YPAG) agreed to collaborate throughout the study. This will include specific input regarding: (i) the recruitment strategy (eg, use of incentives, first approach); (ii) study materials (eg, invitation letters, topic guides, workshop activities and plans); (iii) an initial strategy for multicomponent intervention and (iv) communication materials development. The later meetings will focus on how to disseminate and translate the research outputs to ensure reach with both the target users and key stakeholders. Members of the PPI advisory groups will be invited to co-present study findings at local events.

### Recruitment

#### Phase

We have received preliminary agreement from two immunisation teams in London and the south-west of England to facilitate recruitment. Parents who have not provided consent for their adolescent child to receive the HPV vaccine will be identified through the records of the immunisation teams. There are two distinct population groups represented: (i) parents who have not returned the consent form to the school (passive refusal) and (ii) parents who have completed a consent form refusing the vaccine (active refusal).

At this stage, we hypothesise that the barriers to vaccination uptake may differ between these two groups. For example, passive refusers may be more likely to respond to an intervention that increases accessibility of the vaccine, whereas active refusers may require a communication-based intervention to alter vaccine beliefs. This issue will be explored in detail during the preliminary qualitative study. The findings of which will then be used to guide the co-development of the multicomponent intervention that is most likely to address the barriers to vaccination uptake for the adolescent children of both of these groups.

We aim to recruit a sample of 40 parents, comprising 20 in Bristol and 20 in London. As with any qualitative research, our aim is not to draw definitive conclusions regarding differences by different groups, but rather to capture the breadth of perspectives.

Based on our collective experience of undertaking qualitative research about the HPV vaccination programme, we are confident that we will detect the breadth of different views and perspectives according to different sociodemographic characteristics sufficiently through 40 interviews. The sample size is not based on a formal power calculation, but on our previous experience of undertaking qualitative studies^11 16^ and a pragmatic assessment of the numbers required to obtain sufficient data for us to reach data saturation, detect overarching themes and examine similarities and differences within those themes.

At this stage, we do not believe that further interviews would generate further data required for this preliminary qualitative study. However, we will remain flexible in our approach and, if considered necessary, we will increase the sample size if it becomes apparent that this would be of benefit in terms of gathering sufficient data.

We will aim to recruit to the study a purposive sample of parents to the study based on their gender, ethnicity and whether they actively or passively refused vaccination for their adolescent child. We will recruit participants until ‘data saturation’ has been reached (eg, no new data or information emerges during interviews). This will provide us with confidence that the sample size has been sufficient to capture the breadth of views.

If the representation of parents from minority ethnic groups or more deprived communities is lower than expected, we will draw on our relationships with project partners to facilitate approaches to relevant community organisations to enable recruitment. Information explaining the study and what participation would involve will be provided to potential participants.

Recruitment of professionals (commissioners, healthcare managers, immunisation nurses) (n=8) with different levels of experience will be undertaken through existing relationships between the study researchers and participating healthcare organisations. Through their knowledge and experience of working with families to gain consent for vaccination programmes, healthcare professionals are well placed to offer insight into the feasibility and acceptability of potential interventions. Their understanding of the reasons why parents may not provide consent for vaccinations will also be of benefit to shaping the intervention.

Using existing relationships with a diverse range of community groups (developed in previous research), we will network out to widen the participant pool in collaboration with our key stakeholders.^17–19^ For example, the managers of immunisation teams will be asked to approach relevant team members and contacts within their professional network with different levels of experience in delivery.

#### Phase 2

Parents who took part in the interview study will be approached by the study researcher to take part in workshops and/or filming as part of the co-design study. They will be given information packs about the study. We will aim to recruit eight parents to this part of the study.

### Data collection

#### Phase 1

Interviews will take place either in English or their preferred language facilitated through a translator with appropriate language skills. Separate topic guides for parents and healthcare professionals will be designed in collaboration with the parent advisory groups to ensure relevance to our target population and maximise engagement.

First, participants will be encouraged to discuss their own experiences of the HPV vaccination programme, including factors influencing their decision and reasons for not providing consent. Participants will be asked to: (i) review existing communication materials targeting parents; (ii) comment on their views and understanding of key vaccine messages; (iii) make suggestions of their preferred messages, design and language style that align with their cultural values, beliefs and behaviours; (iv) propose delivery providers of vaccine messages (eg, pharmacists, adolescent children, healthcare professionals, schools); (v) suggest platforms to distribute communication materials (eg, community advocates, general practices, one-to-one with healthcare professionals, web-based forums, interactive information sessions) and (vi) comment on the acceptability of approaches for healthcare professionals to address vaccine hesitancy or refusal.

Participants will be asked to recommend strategies most likely to improve access where parents do not provide consent for the HPV vaccination to be given at the scheduled school-based session (eg, additional mop-up session in school, recall by general practice, availability of the vaccine at pharmacies, community organisations). The data will be scrutinised to identify whether responses differ by ethnicity or type of vaccine refusal (active or passive).

The findings from the qualitative study will be combined with existing literature on increasing vaccine confidence and PPI to inform the development and optimisation of the preliminary intervention plan.^14^

#### Phase 2

At least two working groups comprising parents in London (n~4) and Bristol (n~4) who took part in the qualitative study will be convened to codesign the communication materials, as well as the guiding principles and logic modelling underpinning the intervention.^14^ We will ensure that the working groups include parents from diverse ethnic backgrounds. At least two workshops will be undertaken with each parent group either online or face-to-face depending on their preferences. Workshops will take place either in English or their preferred language facilitated through a translator with appropriate language skills.

We will use an iterative process of collecting data, moving between data collection, analysis, modifications to the strategy and then further data collection. Initially, the adapted plan for the multicomponent intervention will be presented to the group for feedback. Interventions aiming to improve uptake of the HPV vaccine developed from the qualitative research and the literature will be presented to the group to generate further discussion. An ‘ideas group’ approach will be used^20^ where participants contribute ideas most likely to lead to increases in confidence about, and access to, the HPV vaccine. The intervention plan will be iteratively redesigned by considering participants’ views at all stages of the development cycle.

The proposed plan for developing the communication materials will then be presented to the group. Participants will be asked to discuss their preferences for use of language, imagery and tone for the communication materials. Creative approaches to obtaining feedback will be used (eg, post-it note activities, ballot box exercises). Suggested platforms to distribute the communication materials will be confirmed by the group.

Initial communication materials will be developed in collaboration with parents at the working groups and a creative designer experienced in developing health communication materials. These may comprise a series of short films involving members of the working groups or animations, or web-based materials to be distributed on relevant internet pages or through social media campaigns.

The materials will focus on increasing parents’ vaccine confidence, and may focus on addressing parents’ concerns about effectiveness, side-effects, safety profile, content of the HPV vaccine and perceptions of need for their adolescent children. To ensure the content is evidence-based, the most up-to-date information related to safety and side-effects will be gathered from a Cochrane Systematic Review (currently in progress^21^). The content will be tailored, and translated to appropriate languages where needed, to communicate the key messages of the HPV programme while also meeting the needs of the participating communities.

Once an initial prototype for the communication materials has been developed, the same participants will be invited to a second workshop (one held in London and one in the south-west of England) where they will be asked to provide feedback on the language, imagery and tone. Modifications will focus on increasing the acceptability, feasibility and persuasiveness of the communication materials at improving parents’ vaccine confidence.

Key stakeholders (eg, academics, professionals within national immunisation operations, members of immunisation teams, community leaders), the parent PPI community groups in the south-west of England and London, and the Bristol YPAG involved in the earlier stages of the study will be consulted. Participants will be asked to provide feedback on the revised intervention plan and communication materials, including positive and negative aspects, how it was presented, the design, and suggesting or creating new content.

### Analysis

All interview/workshop recordings will be audio recorded using an encrypted digital recorder, transcribed verbatim and any potentially identifying information removed. Transcripts will be checked against the original recordings, corrected as necessary and anonymised. Familiarisation with the data will involve the research team (HF, TC, SD) reading and discussing the transcripts to compare and begin to code and categorise the data. Thematic analysis^22^ will be undertaken, assisted by QSR NVivo V.12 software package. Overarching themes will be identified within which similarities and differences will be explored.

Together, the findings from the qualitative research and co-design study will support the development of the logic model and theoretical underpinning of the targeted, multicomponent intervention using the principles of the ‘person-based approach’.^14^ The outcome from this participatory research will include recommendations for a larger scale study (either a feasibility study, an internal pilot of a full-scale study or a natural experiment).

The proposed outcome measures for a future study will seek to address whether the intervention can: (i) improve uptake of the HPV vaccination programme among young people whose parents did not provide consent for vaccination at the schools-based session; (ii) increase parental HPV vaccine confidence and (iii) increase healthcare professional confidence to recommend the HPV vaccine.

The educational package we have already coproduced with young people^12^ and this current research could in the future complement each other and contribute to a system-wide approach to improving communication about, and increasing uptake of, adolescent vaccination programmes.

## Ethics and dissemination

The National Health Service Research Ethics Dommittee (reference: 22/SW/0003) and London School of Hygiene and Tropical Medicine Observational / Interventions Research Ethics Committee (26902) provided approvals for the study. The project began recruitment in February 2022 and the research activities will finish in March 2023. Informed written or verbal consent will be obtained from parents and key professionals prior to participating in an interview, workshop or filming as part of the study.

The final dissemination plan will be co-designed with members of the PPI groups. In collaboration with our PPI groups, we will summarise the results to share with parents who took part in the study. We will translate the findings of the study to appropriate languages identified during the course of the project. We will also copresent our findings with parents who were involved in codesigning the intervention at community and stakeholder dissemination events.

We will also collaborate with the UK Health Security Agency to organise a webinar with the National Immunisation Network to share the findings more widely with national policy makers. The findings will be presented at national and international academic conferences and at least two manuscripts will be written for submission to peer-reviewed academic journals.

## Supplementary Material

Reviewer comments
