# Supplementary material for: Development of a multicomponent intervention to increase parental vaccine confidence and young people’s access to the universal HPV vaccination programme in England: protocol for a co-design study
Source: BMJ Open. 2022 Apr 6;12(4):e062050. doi: 10.1136/bmjopen-2022-062050 (PMC8987754; doi:10.1136/bmjopen-2022-062050)
Supplement: Reviewer comments [file bmjopen-2022-062050.reviewer_comments.pdf]

PEER REVIEW HISTORY

BMJ Open publishes all reviews undertaken for accepted manuscripts. Reviewers are asked to complete a checklist review form (**http://bmjopen.bmj.com/site/about/resources/checklist.pdf**) and are provided with free text boxes to elaborate on their assessment. These free text comments are reproduced below.

ARTICLE DETAILS

**TITLE (PROVISIONAL)**	Development of a multicomponent intervention to increase parental vaccine confidence and young people's access to the universal HPV vaccination programme in England: protocol for a co-design study
**AUTHORS**	Fisher, Harriet; Chantler, Tracey; Denford, Sarah; Finn, Adam; Hickman, Matthew; Mounier-Jack, Sandra; Roderick, Marion; Tucker, Leanne; Yates, Julie; Audrey, Suzanne

This article was not externally reviewed at BMJ Open. Protocols that have been independently assessed prior to submission to BMJ Open are usually fast-tracked to publication on the grounds that further substantial changes will not be possible. This independent assessment will usually be external, independent review for both a substantial grant award from a non-commercial or government funder and ethics approval.
